# The Spectrum of *ACAN* Gene Mutations in a Selected Chinese Cohort of Short Stature: Genotype-Phenotype Correlation

**DOI:** 10.3389/fgene.2022.891040

**Published:** 2022-05-10

**Authors:** Su Wu, Chunli Wang, Qing Cao, Ziyang Zhu, Qianqi Liu, Xinyan Gu, Bixia Zheng, Wei Zhou, Zhanjun Jia, Wei Gu, Xiaonan Li

**Affiliations:** ^1^ Department of Endocrinology, Children’s Hospital of Nanjing Medical University, Nanjing, China; ^2^ Nanjing Key Laboratory of Pediatrics, Children’s Hospital of Nanjing Medical University, Nanjing, China; ^3^ Department of Child Health Care, Children’s Hospital of Nanjing Medical University, Nanjing, China; ^4^ School of Pediatrics, Nanjing Medical University, Nanjing, China

**Keywords:** ACAN gene, short stature, variants, genotypes and phenotypes, genetic analysis

## Abstract

**Objective:** Mutations in the *ACAN* gene have been reported to cause short stature. However, the prevalence estimates of pathogenic *ACAN* variants in individuals with short stature vary, and the correlation between *ACAN* genotype and clinical phenotype remain to be evaluated. To determine the prevalence of *ACAN* variants among Chinese people with short stature and analyze the relationship between genotype and main clinical manifestations of short stature and advanced bone age among patients with *ACAN* variants.

**Methods:** We performed next-generation sequencing-based genetic analyses on 442 individuals with short stature. *ACAN* variants were summarized, previously reported cases were retrospectively analyzed, and an association analysis between genotype and phenotype was conducted.

**Result:** We identified 15 novel and two recurrent *ACAN* gene variants in 16 different pedigrees that included index patients with short stature. Among the patients with *ACAN* variants, 12 of 18 had advanced bone age and 7 of 18 received growth hormone therapy, 5 (71.4%) of whom exhibited variable levels of height standard deviation score improvement. Further analysis showed that patients with *ACAN* truncating variants had shorter height standard deviation scores (*p* = 0.0001) and larger bone age–chronological age values (*p* = 0.0464). Moreover, patients in this Asian population had a smaller mean bone age–chronological age value than those that have been determined in European and American populations (*p* = 0.0033).

**Conclusion:** Our data suggest that *ACAN* mutation is a common cause of short stature in China, especially among patients with a family history of short stature but also among those who were born short for their gestational age without a family history. Patients with truncating variants were shorter in height and had more obvious advanced bone age, and the proportion of patients with advanced bone age was lower in this Asian population than in Europe and America.

## Introduction

Short stature is defined as a height below the third percentile or more than two standard deviations below the corresponding mean height for those of the same age, sex, and race, and it is among the most common causes of referral to pediatric endocrinologists (Dauber et al., 2014; Argente, 2016). Previous studies have shown that linear bone growth is determined by the growth and division of matrix-producing chondrocytes at the growth plate. The rate of growth plate chondrogenesis is regulated by many factors, such as multiple hormones, paracrine factors, intracellular proteins, and extracellular matrix molecules (Baron et al., 2015). Short stature can be caused by decreased chondrogenesis due to variants in any gene that directly or indirectly affects growth plate chondrocytes and the process of growth plate chondrogenesis (Baron et al., 2015). With the development of genetic testing, about 200 genes have been identified as being involved in growth plate homeostasis (Wit et al., 2016). In recent years, genes involved in extracellular matrix maintenance, such as *ACAN*, *FBN1*, *COMP*, *ADAMTS10*, *COL10A1*, *COL9A1*, *COL9A2*, and *COL9A3* have attracted more attention (Wit et al., 2016), especially *ACAN* (Nilsson, 2020; Stavber et al., 2020; Lin et al., 2021).

As one of the most abundant molecules in the extracellular cartilage matrix, aggrecan plays a key role in the morphogenesis of cartilage and bone, and *ACAN* gene variation is a major cause of short stature (Nilsson et al., 2014; Sentchordi-Montané et al., 2018; Lin et al., 2021). Homozygous *ACAN* variants lead to spondyloepimetaphyseal dysplasia, aggrecan type (SEMD, OMIM#612813) (Tompson et al., 2009). Heterozygous variants can cause spondyloepiphyseal dysplasia, Kimberley type (SEDK, OMIM#608361) (Gleghorn et al., 2005) or short stature and advanced bone age, with or without early-onset osteoarthritis and/or osteochondritis dissecans (SSOAOD, OMIM#165800) (Stattin et al., 2010; Tatsi et al., 2017). With increasing focus on aggrecan in relation to understanding the physiologic mechanisms underlying short stature in recent years, many individuals with *ACAN* variants have been reported. The frequency of *ACAN* variants among individuals of short stature range from 1.2% to 37.5% (Hauer et al., 2017; Hu et al., 2017; Plachy et al., 2019; Stavber et al., 2020; Lin et al., 2021). The initial studies found that the main clinical manifestations of patients with *ACAN* variants are short stature with advanced bone age (Nilsson et al., 2014; Quintos et al., 2015). Later, other individuals who presented with short stature and normal or even delayed bone age were reported (Hu et al., 2017; Stavber et al., 2020; Lin et al., 2021). Although previous studies have investigated bone age differences among *ACAN* variant carriers of different races (Lin et al., 2021), no correlations between genotype and phenotype have been found (Dateki, 2017; Gkourogianni et al., 2017; Liang et al., 2020).

In this study, we screened 442 children of short stature and found *ACAN* variants in 16 probands. We analyzed the clinical characteristics and mutation spectrum of patients with *ACAN* variants, as well as the effect of growth hormone (GH) therapy on these individuals. Additionally, based on the patient data reported in this article and to date, we also performed an association analysis between genotype and phenotype.

## Materials and Methods

### Study Patients

At our center, 442 children with short stature underwent exome sequencing. We enrolled 95 children who were tested because they were born small for gestational age (SGA), 143 children with familial short stature, 60 children with both SGA and familial short stature, and 144 children of short stature complicated with other features, such as intellectual disability, facial abnormalities, skeletal malformations, and other systemic diseases. The detailed workflow of patient selection is summarized in Supplementary Figure S1. All study participants or their legal guardians signed written informed consent forms before exome sequencing was carried out. The study was approved by the institutional ethics committee of Children’s Hospital of Nanjing Medical University.

### Clinical and Laboratory Data

The clinical data included age at diagnosis, phenotypic appearance, family history, physical characteristics (height, weight, body mass index, etc.), bone age, and radiographic changes of the spine. The biochemical data included IGF1 levels and peak GH levels from the GH stimulation test. More detailed methods regarding biochemical assessment are included in the Supplementary Methods. Height measurement was used to evaluate growth and converted to a standard deviation score (SDS) using standardized growth charts for Chinese children and adolescents aged 0–18 years (Li et al., 2009).

### Treatment and Follow-Up

After thorough communication with the children and their guardians, we gave recombinant human GH (rhGH) therapy to children who were willing to receive rhGH treatment, and the initial dose was recommended mainly based on the results of the GH stimulation test according to current guidelines (The Subspecialty Group of Endocrinologic and Hereditary and Metabolic Diseases, 2008), More details are included in the Supplementary Methods. For children with pubertal development, a combination of gonadotropin-releasing hormone agonists or aromatase inhibitors was considered. Every 3–6 months, we documented changes in height SDS, laboratory data (including serum biochemistry, blood glucose, thyroid function, IGF1, and bone age) from the time of diagnosis to the time of inclusion in the study.

### Genetic Analysis

Exome sequencing was performed as previously described (Wang et al., 2020). Briefly, Genomic DNA was extracted from peripheral blood using a DNA isolation kit (Tiangen, Beijing, China) and subjected to whole-exome sequencing (WES) and targeted next-generation sequencing (a list of 286 short related genes is shown in Supplementary Table S1) on the Illumina HiSeq 2500 sequencing platform. The obtained sequences were aligned to a reference human genome (hg19 build) using BWA software. Single nucleotide variation (SNV), inserts and deletions (INDEL) were filtered by GATK software (https://software.broadinstitute.org/gatk/). Then all variants were further annotated by ANNOVAR software. The variant sites with frequencies more than 1% in the public databases [Genome Aggregation Database (gnomAD), dbSNP, 1000 Genomes MAF (Chinese), ExAC, and an in-house MAF database] were removed. After the above steps, missense variants were predicted by SIFT, PolyPhen-2, MutationTaster and GERP++, pathogenic forecasts, and conservative projections, while splice sites were predicted by three web-based programs: Alternative Splice Site Predictor, Human Splicing Finder Version 3.0, Splice Site Prediction by Neural Network. The primer sequences are available upon request. All candidate variants were clarified in accordance with the American College of Medical Genetics and Genomics (ACMG) criteria (Richards et al., 2015) and further validated by Sanger sequencing.

### Review of Published *ACAN* Variants

The terms “*ACAN*” and “variants” or “mutations” were used to search for articles reporting on individuals of short stature in PubMed. Additionally, variants present in the Human Gene Mutation Database (HGMD) professional database (Biobase, Qiagen) were reviewed. The accuracy of variant description was checked using Alamut 2.10 (Interactive Biosoftware). Deletions encompassing *ACAN* and additional genes were excluded. *ACAN* variants are indicated on the longest isoform (complementary DNA: NM_001135.4; protein: NP_001126.3) unless specified differently, according to Human Genome Variation Society (HGVS) guidelines (www.hgvs.org/mutnomen). Pathogenic variants and corresponding patient data (sex, status, variant inheritance, height SDS, clinical manifestations, biochemical, and radiological characteristics) were listed (Supplementary Table S2) and visualized on the schematic representation of the *ACAN* gene.

### Genotype-Phenotype Correlation

The data considered for the genotype-phenotype correlation in the probands were: bone age and height SDS. Genotypes were subdivided into two groups. One group consisted of patients with truncating mutations (TMs), including stop mutations, insertions and deletions causing a frameshift and a premature stop codon, splice-site mutations, and exon deletions leading to a truncated protein. The second group contained patients with missense mutations categorized as the nontruncating mutations (NTMs) group.

### Statistical Analysis

The biochemical characteristics for all patients are presented as mean ± SD. Data were analyzed with the use of the statistical packages R (R Foundation for Statistical Computing, Vienna, Austria; http://www.r-project.org; version 3.4.3), Empower (R) (www.empowerstates.com, X&Y Solutions, Inc. Boston, MA, United States) and GraphPad Prism 5 (GraphPad Software, Inc., San Diego, CA, United States). Between-group comparisons were performed using nonparametric tests. Two-tailed *p* values < 0.05 were considered statistically significant.

## Results

### Variants Detected in *ACAN*

**FIGURE 1:**
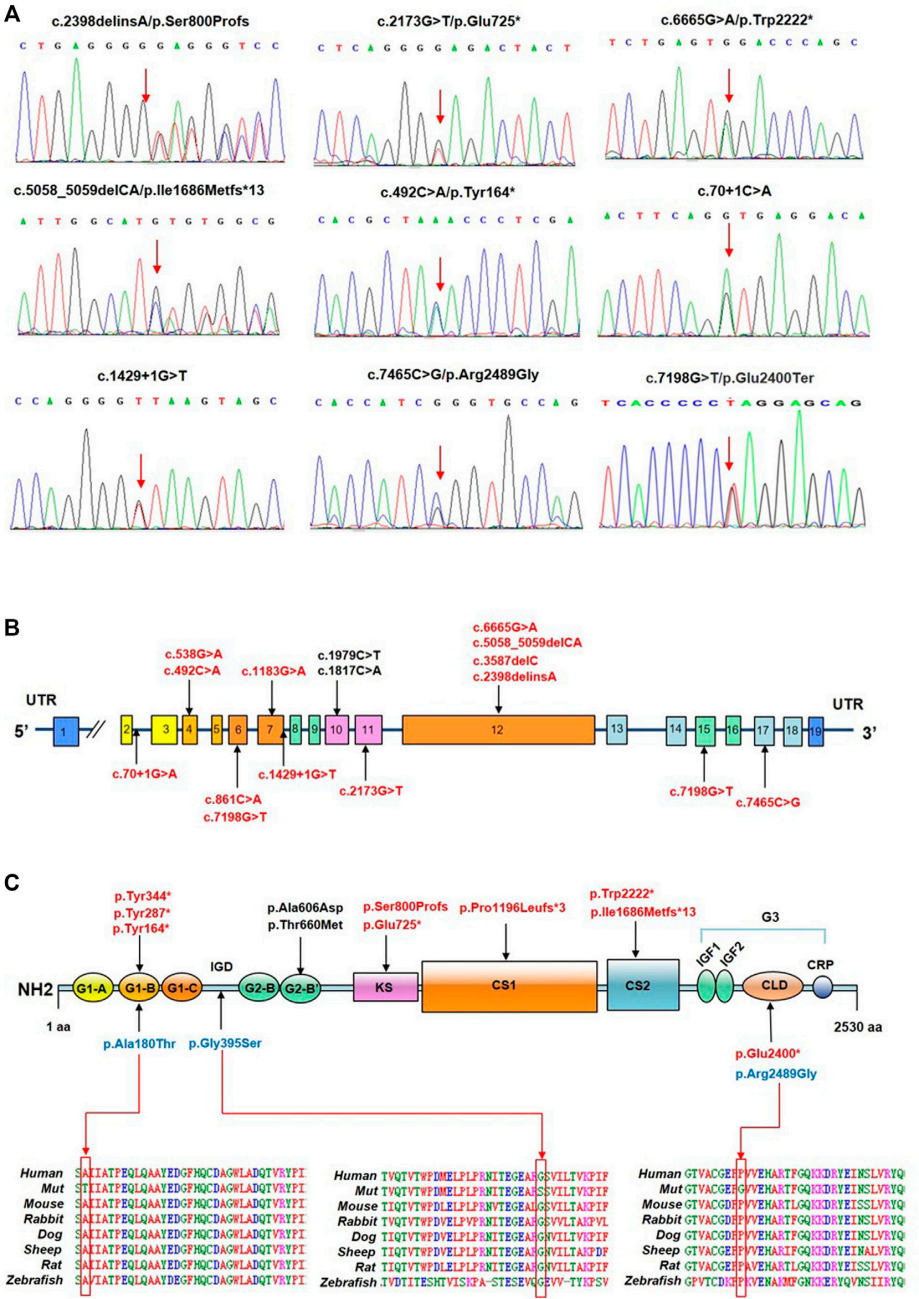
Identification of *ACAN* variants in patients with short stature. **(A)** Sequence chromatograms of *ACAN* variants as detected. **(B)** Distribution of *ACAN* variants in exons and introns. **(C)** *ACAN* mutations are located in corresponding domains. Abbreviations: G1, globular domain 1; IGD, interglobular domain; G2, globular domain 2; KS, keratan sulfate; CS1, chondroitin sulfate 1; CS2, chondroitin sulfate 2; G3, globular domain 3; EGF1, 2, epidermal growth factor-like domain 1, 2; CLD, C-type lectin domain; CRP domain, complement regulatory protein-like domain. The red arrow is pointing at orthologous and paralogous protein alignments showing the high conservation of each amino acid altered by three missense variants from humans to zebrafish.

A total of 17 *ACAN* variants (six nonsense, five missense, three frameshift, two splice site, one deletion) were identified in the 16 different pedigrees (Table 1; Figure 1). Among them, there were 15 novel variants including five missense variants [(c.538G > A (p.Ala180Thr), c.1183G > A (p.Gly395Ser), c.1817C > A (p.Ala606Asp), c.1979C > T (p.Thr660Met) and c.7465C > G (p.Arg2489Gly)], six nonsense variants [(c.492C > A (p.Tyr164*), c.861C > A (p.Tyr287*), c.1032C > G (p.Tyr344*), c.2173G > T (p.Glu725*), c.6665G > A (p.Trp2222*), c.7198G > T (p.Glu2400*)], three frameshift variants [(c.2398delinsA (p.Ser800Profs), c.3587delC (p.P1196Lfs*3), and c.5058_5059delCA (p.I1686Mfs*13)], one deletion (del exon12) (Supplementary Figure S6), and two splice site variants (c.70+1G > A and c.1429+1G > T). Three missense variants (p.Ala180Thr, p.Gly395Ser, and p.Arg2489Gly) showed high conservation of each amino acid altered from humans to zebrafish (Figure 1C). The two novel splice site c.1429+1G > T, c.70+1G > A variants on pre-mRNA splicing was confirmed *in vitro* using a minigene (pSPL3 splicing) assay. The c.70+1G > A and c.1429+1G > T variants caused exon2 and exon7 skipping and truncated mRNA in HEK293 cells (Supplementary Figure S2).

**TABLE 1:** Mutations of ACAN gene identified in this study.

Patient	Nucleotide Change	Protein Change	Hom/Het	Location	Domain	Mutation Type	Denovo	ACMG	Phenotype	Reported
P1, P2	c.1817C > A	p.Ala606Asp	Het	E10	G2-B′	Missense	No	UVS (PM2 + PP1)	SSOAOD	Liang et al. (2020)
P3, P4	c.1429+1G > T	-	Het	I7	-	Splicing	No	LP (PVS1 + PM2)	SSOAOD	No
P5	c.538G > A	p.Ala180Thr	Het	E4	G1-B	Missense	No	UVS (PM2)	SSOAOD	No
P6	c.70+1G > A	-	Het	I2	-	Splicing	No	LP (PVS1 + PM2)	SSOAOD	No
P7	c.6665G > A	p.Trp2222*	Het	E12	CS2	Nonsense	No	LP (PVS1 + PM2)	SSOAOD	No
P8	c.5058_5059delCA	p.Ile1686Metfs*13	Het	E12	CS2	Frameshift	No	LP (PVS1 + PM2)	SSOAOD	No
P9	c.492C > A	p.Tyr164*	Het	E4	G1-B	Nonsense	NA	LP (PVS1 + PM2)	SSOAOD	No
P10	c.1032C > G	p.Tyr344*	Het	E6	G1-B′	Nonsense	No	LP (PVS1 + PM2)	SSOAOD	No
P11	c.2398delinsA	p.Ser800Profs	Het	E12	KS	Frameshift	No	LP (PVS1 + PM2)	SSOAOD	No
P12	c.3587delC	p.Pro1196Leufs*3	Het	E12	CS1	Frameshift	No	LP (PVS1 + PM2)	SSOAOD	No
P13	c.2173G > T	p.Glu725*	Het	E11	KS	Nonsense	No	LP (PVS1 + PM2)	SSOAOD	No
P14	c.861C > A	p.Tyr287*	Het	E6	G1-B′	Nonsense	No	LP (PVS1 + PM2)	SSOAOD	No
P15	c.7198G > T	p.Glu2400*	Het	E15	G3	Nonsense	No	LP (PVS1 + PM2)	SSOAOD	No
P16	c.7465C > G	p.Arg2489Gly	Het	E17	G3	Missense	No	UVS (PM2)	SSOAOD	No
P17	Del exon12	-	Hom	E12	-	Deletion	No	LP (PVS1 + PM2)	SEMD	No
P18	c.1979C > T	p.Thr660Met	Het	E10	G2-B′	Missense	No	UVS (PP3 + BS2)	SEMD	Hattori et al. (2017)
P18	c.1183G > A	p.Gly395Ser	Het	E7	IGD	Missense	No	UVS (PP3 + BS2)	SEMD	No

### Clinical Characteristics

**FIGURE 2:**
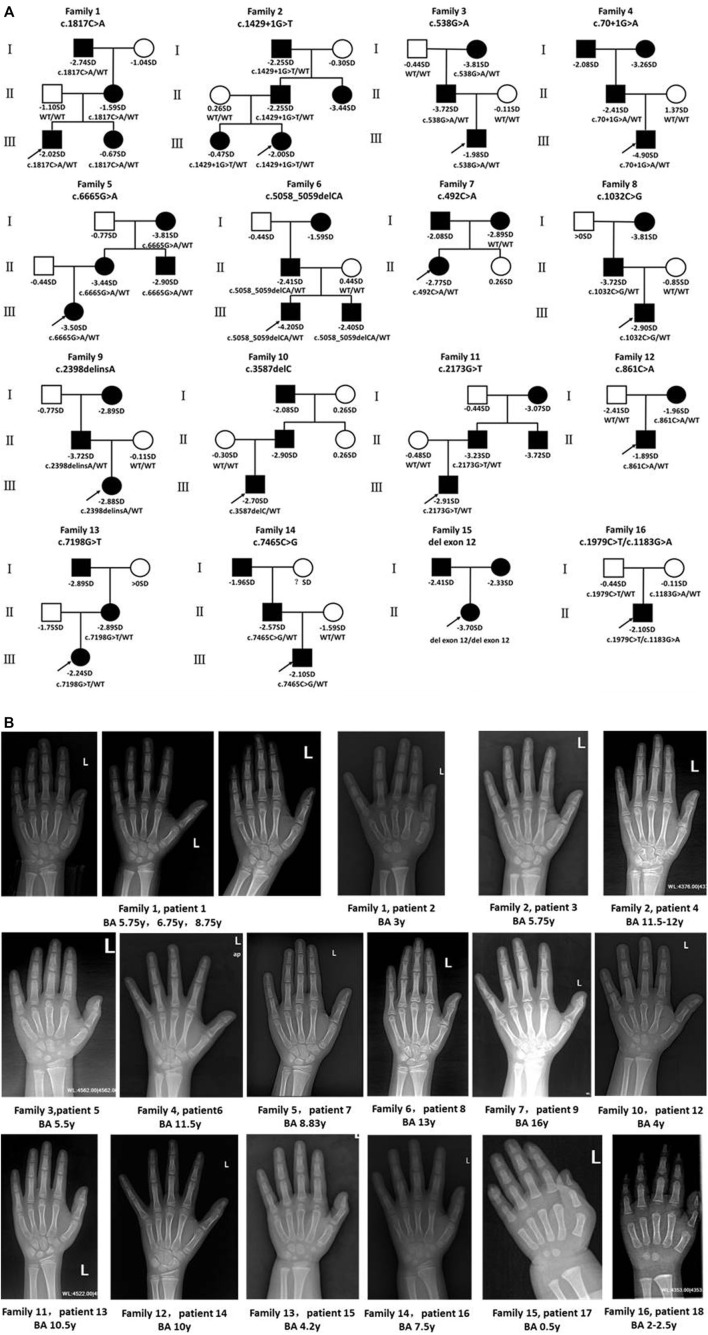
Pedigrees of affected families and hand radiographs of the probands. **(A)** Pedigrees of 16 families with *ACAN* pathogenic variants. Squares indicate males, circles females, filled symbols indicate that the individual presented short stature, open symbols indicate healthy individuals. Probands are denoted by arrows. Abbreviation: ?, unknown phenotype. **(B)** Hand radiographs of individuals carrying *ACAN* variants .

We identified 18 patients (eight girls, ten boys) from 16 different pedigrees who carried *ACAN* gene variants whose ages ranged from 1.33 to 14.25 years (median, 7.26 years). All of the patients were identified from the pediatric endocrinology clinic and had been evaluated for symmetrical short stature, except for P1 and P14 who underwent gene testing because of a low growth velocity (GV < 5 cm/year) with advanced bone age and family history of short stature. Of the aforementioned 16 families, 15 had a family history of short stature; one (P18) had no such family history. Two of the patients (P7, P17) were born SGA, while the other patients were born with normal weights (Table 2; Figure 2A). Ten patients (P1, P3, P5-8, P10, P13, P16, P18) underwent GH stimulation tests. The peak GH level of P18 was 4.339 ng/ml, suggesting complete GH deficiency. The peak GH levels of P6, P8, and P16 were 7.332, 6.219, and 8.46 ng/ml, respectively, suggesting partial GH deficiency. The other six patients showed normal levels of GH (Table 2).

**TABLE 2:** Clinical features of children with ACAN Variants.

Family	Patient	Age (year)	Sex	Nation	Family history	Parental Ht (F/M cm)	BH (cm)	BW (kg)	Birth history	Ht (cm)	Ht SDs	Wt (kg)	BA (year)	BA advanced	Peak GH (ng/ml)	IGF1 (ng/ml)	Tanner
1	P1	6.92	Female	Han	+	166/152	52	3.75	FTCS	118.7	−0.67	24	8.75	Yes	14.213	287	B1P1
	P2	2.75	Male	Han	+	166/152	50	3.4	FTCS	87.4	−2.02	13	3	No	NA	NA	G1P1
2	P3	5.75	Female	Han	+	159/162	50	3.3	FTND	106.5	−2	16.5	5.75	Yes	13.067	203	B1P1
	P4	10.83	Female	Han	+	159/162	50	NA	FTND	142.4	−0.47	41	11.5–12	Yes	NA	NA	B4P2
3	P5	4.5	Male	Han	+	150/160	50	3.3	FTND	99.6	−1.98	18	5.5	Yes	11.414	131	B1P1
4	P6	14.25	Male	Han	+	158/168	50	2.9	FTCS	132.2	−4.9	45	11.5	No	7.332	167	G2P1
5	P7	8.17	Female	Han	+	170/142	NA	2.6	FTND	116.5	−3.5	23	8.83	Yes	12.631	212	B2P1
6	P8	12.92	Male	Han	+	158/163	50	3.35	FTND	137.9	−4.2	46.3	13	Yes	6.219	530	G3P3
7	P9	14	Female	Han	+	160/145	NA	NA	FTCS	132.8	−2.77	45.5	16	Yes	NA	NA	B4P4
8	P10	11.17	Male	Han	+	150/156	NA	NA	NA	126.9	−2.9	33	11	No	>10	NA	B1P1
9	P11	1.33	Female	Han	+	150/160	51	4.45	NA	71.3	−2.88	8	NA	NA	NA	NA	B1P1
10	P12	3.08	Male	Han	+	155/159	50	3.35	FTND	87.2	−2.7	14.2	4	Yes	NA	72.5	B1P1
11	P13	10.08	Male	Han	+	153/158	NA	3.3	FTCS	122.7	−2.91	25.5	10.5	Yes	>34.8	365	B1P1
12	P14	9.75	Male	Han	+	174/148	NA	3.1	FTCS	127.7	−1.77	26.7	10	Yes	NA	417	G1P1
13	P15	3	Female	Han	+	162/145	47	3.38	FTCS	87.3	−2.24	12.3	4.2	Yes	NA	110	B1P1
14	P16	7.25	Malel	Han	+	157/152	50	3.1	FTCS	114.8	−2.1	26	7.5	Yes	8.46	135	B1P1
15	P17	1.5	Female	Han	+	158/148	NA	2.5	FTND	70.5	−3.7	7.65	0.5	No	NA	46.6	B1P1
16	P18	3.5	Male	Han	−	170/160	50	3.9	FTCS	92.6	−2.1	14	2–2.5	No	4.339	58.2	B1P1

Abbreviation: Ht, height; F/M, father/mother; BH, birth height; BW, birth weight; Ht SDs, height standard deviation score; Wt, weight; BA, bone age; FTCS, full-time cesarean section; FTND, full-term normal delivery.

Bone age was determined in 17 patients (P11 excluded), and 12 patients (P1, P3-5, P7-9, P12-16) presented with advanced bone age, while the other patients presented with bone age delay (Table 2; Figure 2B). Spinal X-rays were evaluated for 11 patients (Supplementary Figure S3), with seven patients adjudged to have normal spines (P3, P5, P6, P7, P13, P17, P18). But, the thoracolumbar curve of P1 was slightly straighter than normal, P8 had S1 laminar discontinuity, P9 had cryptomerorachischisis, and P16 had mild scoliosis. X-rays of the hips and knees of P9 and P18 showed no abnormalities. However, X-ray of P18’s left hand revealed abnormal morphology at the proximal ends of the second to fourth metacarpal bones.

### Treatment and Follow-Up Outcomes

Seven of the 18 patients were treated with rhGH and were followed up at our center. Table 3 summarizes the height SDS values related to age at diagnosis and at follow-up consultations for patients who received rhGH treatment. To evaluate the growth response of rhGH treatment, we mainly used height SDS (including the first-year and annualized height SDS) and referred to height velocity. We regarded patients with a first-year delta height SDS larger than 0.3 to 0.5 or a first-year height velocity increase larger than 3 cm/year as good responses (Cohen et al., 2008). Treatment with rhGH was discontinued for P6 and P8 because of poor growth velocity; P6’s treatment lasted only 1.5 months. P8 was treated with rhGH combined with letrozole for 5 months with a height increase of 0.2 SDs. The remaining five patients who were under ongoing treatment and had good responses to GH, particularly P2, who exhibited an increased height of 0.7 SDs in 6 months.

**TABLE 3:** Responses to growth hormone therapy.

Patients	ACAN gene	Age (year)	Ht SDS before	Ht SDS after	Time of treatment(m)	△Ht SDS	△Ht SDS (year)	rhGH (IU/kg/d)
P1	c.1817C > A (p.A606D)	7.0	−0.7	−0.1	18	0.6	0.4	0.17
P2	c.1817C > A (p.A606D)	2.92	−2.1	−1.4	6	0.7	1.4	0.12
P3	c.1429+1G > T (splicing)	6.0	−2	−1.4	12	0.6	0.6	0.16
P5	c.538G > A (p.A180T)	4.53	−2	−0.9	14	1.1	0.9	0.16
P6	c.70+1G > A (splicing)	14.25	−4.9	−5	1.5	−0.1	-	0.13
P8	c.5058_5059delCA (p.I1686Mfs*13)	12.75	−2.7	−2.6	5	0.1	0.2	0.16
P16	c.7465C > G (p.R2489G)	7.42	−2.1	−1.7	9	0.4	0.5	0.13

P8 was treated with rhGH, combined with letrozole 2.5 mg qd.

### Genotype–Phenotype Associations

**FIGURE 3:**
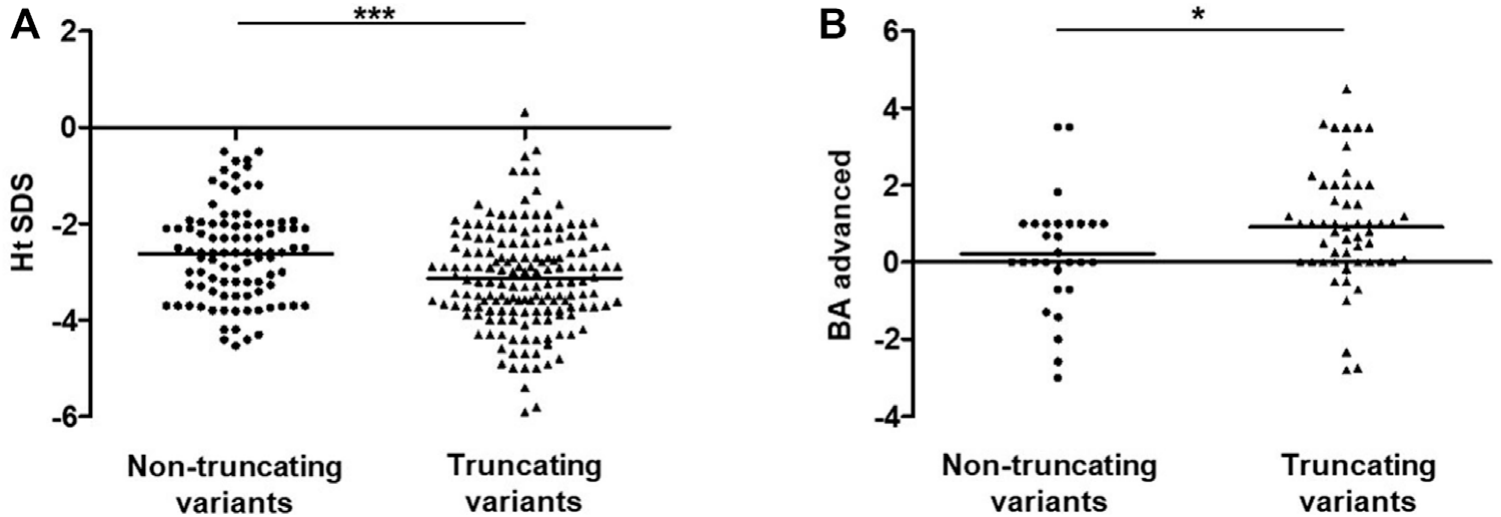
Analysis of Ht SDS and BA-CA between non-truncating variants and truncating variants groups in SSOAOD patients. **(A)** Comparison of Ht SDS between non-truncating variants and truncating variants groups. **(B)** Comparison of BA-CA non-truncating variants and truncating variants groups. *p < 0.05, ***p < 0.001. Abbreviation: Ht SDS: height standard deviation, BA: bone age, CA: chronological age; truncating mutations: frameshift mutations, nonsense mutation, synonymous mutations, splicing mutation, exon deletion; non-truncating mutations: missense mutations, small deletion.

As of 1 April 2021, a total of 76 reported and 15 unreported *ACAN* variants from this study had been summarized, including missense (*n* = 31), frameshift (*n* = 26), nonsense (*n* = 27), splicing (*n* = 5), and deletion (*n* = 2) variants (Supplementary Figure S4). All of the variants were found in 104 families; SSOAOD (*n* = 94) is the main associated phenotype, while SEDK (*n* = 5) and SEMD (*n* = 5) are rare (Supplementary Table S2). Then, 94 probands from 94 SSOAOD families were subdivided into two groups according to genotypes: the TMs (*n* = 62) and NTMs (*n* = 32) groups. Comparisons between the two groups found that probands with TMs have more severe short stature, but this trend was not statistically significant (*p* = 0.089). When we extended the height SDS data to all 258 affected individuals in the 94 SSOAOD families, and the difference in height SDSs was significant (*p* = 0.0001) (Figure 3A). Probands with TMs had more advanced bone age (*p* = 0.046) (Supplementary Table S3; Figure 3B).

Probands were further divided into Asian and non-Asian (European and American) groups, and comparisons between the two groups showed that advanced bone age was more obvious in cases reported in Europe and America (*p* = 0.003) (Supplementary Table S4; Supplementary Figure S5B). In Europe and America, 68.2% (30/44) of population had advanced bone age, compared with only 55.8% (24/43) of the Asian population that had advanced bone age (Supplementary Table S4).

## Discussion

*ACAN* variants have been reported as a cause of short stature, with frequencies of 1.4%–37.5% in short stature populations (Hauer et al., 2017; Hu et al., 2017; Plachy et al., 2019; Stavber et al., 2020; Lin et al., 2021). In a recent study on a large sample of a Chinese idiopathic short stature population, the prevalences of *ACAN* variants among individuals of short stature and a familial subcohort were 1.2% and 3.5%, respectively (Lin et al., 2021). In our study, a total of 16 probands of short stature caused by *ACAN* variants were found among 442 patients of short stature. The prevalences of *ACAN* variants among patients of short stature and the familial subcohort in this study were 3.6% and 7.9%, respectively. This slightly higher proportion may be due to the differences in the selection of the study samples. Previous studies have shown that the prevalence of *ACAN* variants among SGA children was 1.38% (Freire et al., 2019). Similar to previous research, we identified short stature caused by *ACAN* variants among two SGA children with persistent short stature, accounting for 1.3% of our study sample. Our data suggest that *ACAN* gene variation is a major cause of short stature, especially familial short stature, and it is also a cause of SGA with persistent short stature. Meanwhile, complex heterozygous *ACAN* variants can also be found in patients with neither non-familial short stature nor SGA.

Additionally, our study identified 17 different *ACAN* variants in 16 different pedigrees. Fifteen of the 17 variants had not been reported before (Hattori et al., 2017; Liang et al., 2020). Our findings significantly expanded the *ACAN* mutation spectrum. The majority of the ACAN variants (70.6%) were predicted to lead to aggrecan truncation, including nonsense and frameshift mutations, splicing mutations, and large insertions/deletions. Similar to previous reports, our results suggest a high prevalence of *ACAN* truncating variants in the Chinese populaiton (Lin et al., 2021). SSOAOD is the dominant phenotype associated with *ACAN* variation according to the HGMD database. Our study also demonstrated SSOAOD (87.5%) as the predominant phenotype. Although two highly conserved missense variants (p.Ala180Thr and p.Arg2489Gly) in our study were predicted to be VUS according to the ACMG, the patients had typical familial short stature with advanced bone age, so these were considered as pathogenic variants, which should be verified by further sduties. We also identified complex heterozygous mutations (c.1979C > T/c.1183G > A) in patient 18, the proband presented with short stature, combining with cartilage damage, which was consistent with the characteristics of *ACAN* mutations related phenotype. It's worth noting that, the c.1979C > T has been previously reported to be associated with SSOAOD, however, the proband’s father did not have short stature, suggesting the possibility of explicit incompleteness. The c.1183G > A/p.Gly395Ser change is located within IGD domain of the protein. This variant yielded predominantly deleterious prediction scores by three algorithms (PolyPhen-2, MutationTaster and SIFT). The conservation of the Gly residue in position 395 across evolution shows it is well conserved from *Homo sapiens* to zebrafish (Roughley and Mort, 2014).

We summarized *ACAN* variants that have been associated with short stature both in previous reports and our study. A total of 91 *ACAN* variants were included; missense, frameshift, and nonsense variants were the main variant types, with splicing and deletion variants being relatively rare. All the variants were found in 104 families; SSOAOD was the main associated phenotype, while SEDK and SEMD were rare. Although previous studies suggested that the severity of short stature was not associated with variant types (Lin et al., 2021), and no correlations between genotype and phenotype had been found (Dateki, 2017; Gkourogianni et al., 2017; Liang et al., 2020), we found that individuals with truncating variants had more severe short stature. Our study identified a correlation between genotype and phenotype in terms of height among individuals with *ACAN* variants. Although the mechanism is not clear, it may be related to the greater effect of truncating variants on ACAN protein function. Previous studies have shown that most truncating variants in *ACAN* lead to premature stop codons, which may result in early truncation and probably nonsense mediated mRNA decay, implying haploinsufficiency is probably the mechanism in patients with *ACAN* mutations (Perchard et al., 2020; Lin et al., 2021). Combing with minigene assay confirmation *in vitro*, we found that patients with severe truncating variants had more severe short stature in our cohort, which is consistent with the haploinsufficiency mechanism mentioned above.

Besides the differences in severity of short stature found in cases with different variants, we also demonstrated that cases with truncating variants had more advanced bone age. Differences in bone age characteristics have been found across populations in previous studies (Lin et al., 2021); similarly, our study showed that advanced bone age was more obvious in cases reported in Europe and America, 68.2% (30/44) of the European and American population showed advanced bone age, while only 55.8% (24/43) of the Asian population showed advanced bone age. Our study identified the correlation between genotype and phenotype in terms of advanced bone age in patients with *ACAN* variants. The true nature of the effect of *ACAN* variation on bone age has not been elucidated. Previously, research has shown that the IHH, FGF, and BMP signaling pathways are altered from the very beginning of growth plate formation in the absence of aggrecan, which induced premature hypertrophic chondrocyte maturation (Domowicz et al., 2009) and might be related to advanced bone age. However, advanced bone age is a complicated issue that requires further study; likewise, the differences across populations may be related to the difference in age, sexual development and enrollment conditions between two groups, and warrant further study in groups with larger samples.

Although short stature and advanced bone age are the main clinical features of patients with *ACAN* variants, there are other associated characteristics, such as facial hypoplasia, short neck, brachydactyly, short thumbs, short metacarpal bones, short limbs, lumbar lordosis, and scoliosis (Quintos et al., 2015; Dateki et al., 2017; Sentchordi-Montané et al., 2018; Liang et al., 2020). Due to the lack of detailed descriptions of the clinical characteristics mentioned above in some reported research, as well as in the present study, correlation analysis between such clinical phenotypes and genotypes was not further conducted, and further sample expansion and detailed study are required in the future.

To date, GH remains the primary treatment for improving height for patients with *ACAN* variants, and combination therapy with aromatase inhibitors or gonadotropin-releasing hormone antagonists may be needed for adolescent patients with advanced bone age. A previous study found that half of 26 treated individuals showed moderate to good responses to GH, but the findings represented poor responses after the age of 10 years (Gkourogianni et al., 2017; Hauer et al., 2017; Tatsi et al., 2017; van der Steen et al., 2017; Xu et al., 2018; Lin et al., 2021). Our study also showed that all patients younger than 10 years responded well to rhGH therapy, and the younger the age at the initiation of treatment, the better the effect of rhGH treatment. Therefore, for patients with *ACAN* gene variants, early administration of GH is associated with more marked height improvements.

## Conclusion

In conclusion, we identified *ACAN* gene variation as the main pathogenic factor in a Chinese cohort of children of short stature; this was especially true for individuals with familial short stature. Moreover, we reported 15 novel variants and expanded the *ACAN* variant spectrum. In all reported cases, we found that patients with truncating variants were shorter in height and had more obvious advanced bone age, and the proportion of patients with advanced bone age was lower in our Asian population than in Europe and America. Finally, this study also verified the short-term efficacy of GH therapy for patients with *ACAN* gene variants, but the long-term efficacy needs further study.

## Data Availability

The datasets for this article are not publicly available due to concerns regarding participant/patient anonymity. Requests to access the datasets should be directed to the corresponding authors.
