# Supplementary material for: The Spectrum of ACAN Gene Mutations in a Selected Chinese Cohort of Short Stature: Genotype-Phenotype Correlation
Source: Front Genet. 2022 May 10;13:891040. doi: 10.3389/fgene.2022.891040 (PMC9127616; doi:10.3389/fgene.2022.891040)
Supplement: Supplementary file 1 [file DataSheet4.DOCX]

**Supplementary Figures**

**
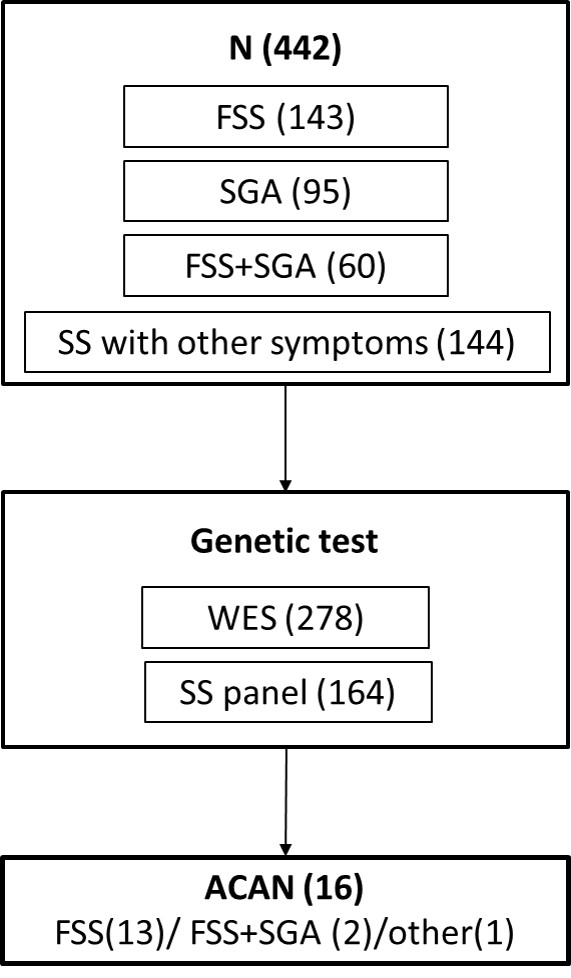
**

**Fig S1. Work flow summarizing the selection of patients for the study and the genetic results.**

**
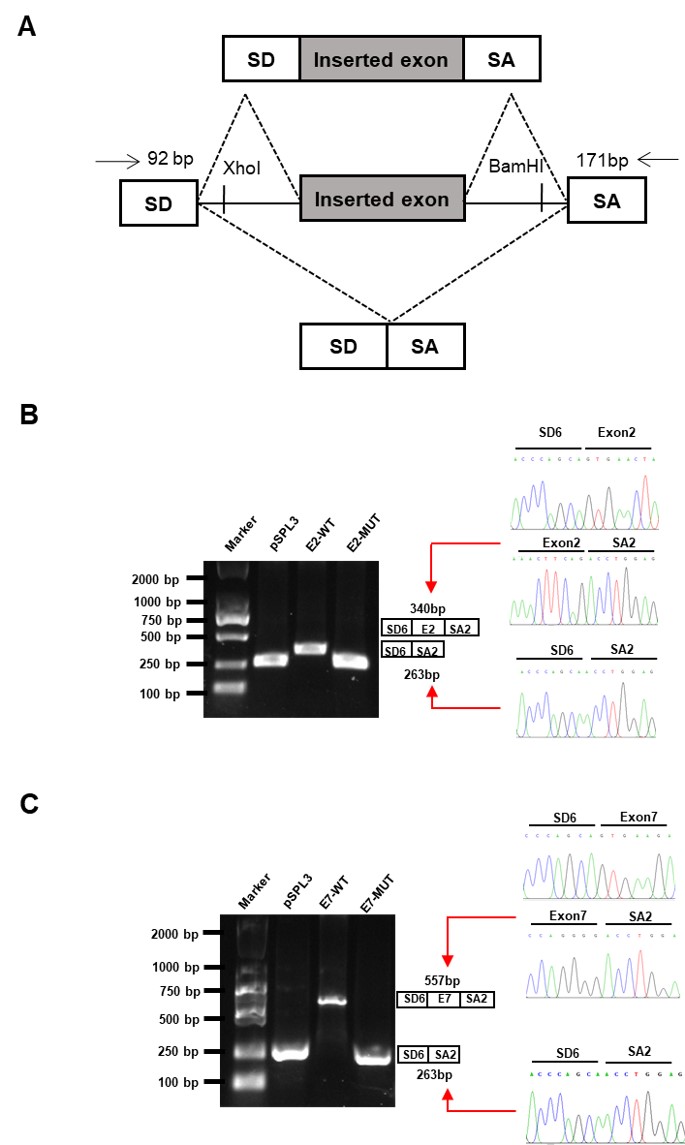
**

**Fig S2. Effect of ACAN gene c.70+1G>A and c.1429+1G>T variants determined by minigene assays.** (A) The transcripts produced by the hybrid minigene are shown schematically. (B) Gel electrophoresis of the RT–PCR products of minigene transcripts in HEK293 cells. Lane 1: marker; Lane 2: pSPL3 (263bp); Lane 3: E2-WT (340bp); Lane 4: c.70+1G>A (340bp). The two fragments were directly sequenced (right panel). (C) Gel electrophoresis of the RT–PCR products of minigene transcripts in HEK293 cells. Lane 1: marker; Lane 2: pSPL3 (263bp); Lane 3: E2-WT (557bp); Lane 4: c.1429+1G>T (263bp). The two fragments were directly sequenced (right panel).


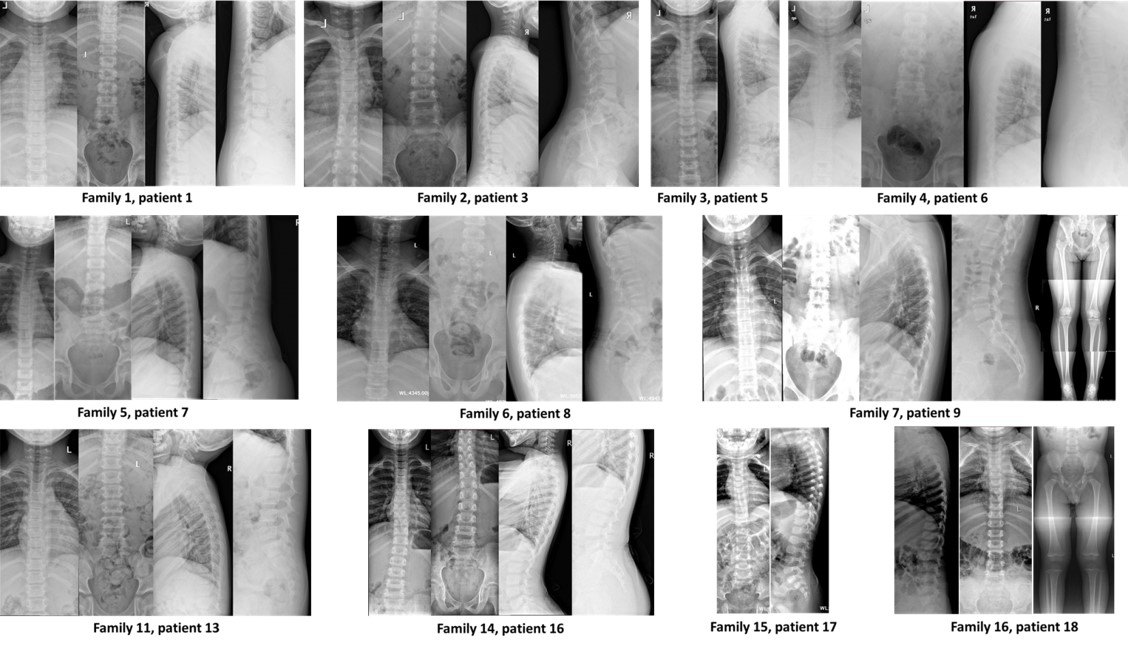


**Fig S3. Radiographs of some affected patients carrying ACAN variants.**

**
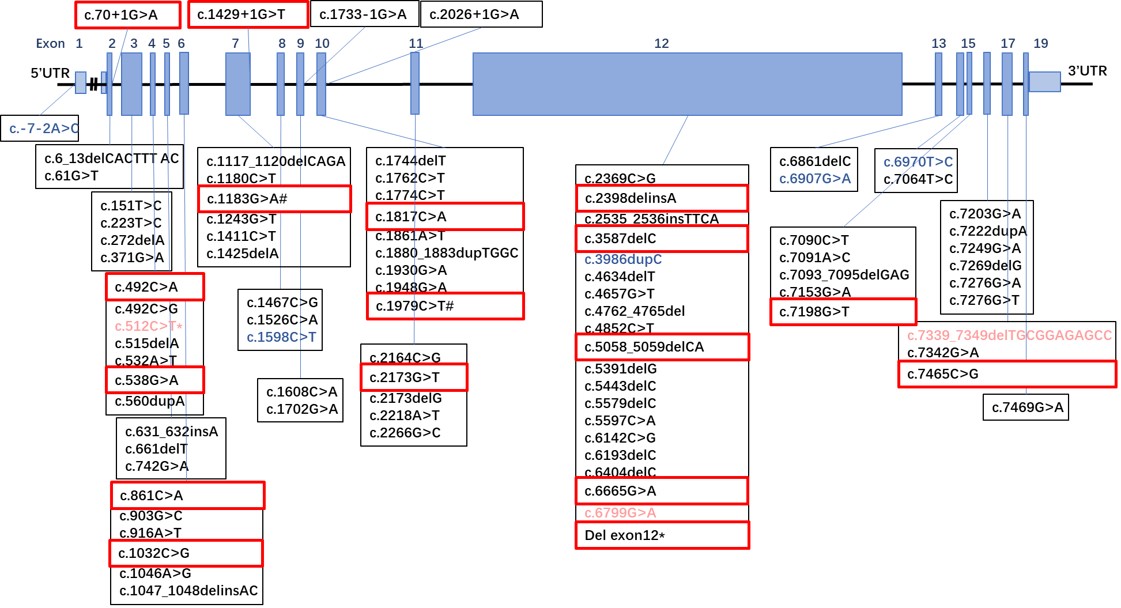
**

**Fig S4. Schematic of the ACAN gene and the locations of reported pathogenic variants.** Variants with black font：SSOAOD; variants with pink font: SEMD; variants with blue font: SEDK. Mutations with red border are from this study. Abbreviation: # Complex heterozygous mutation of c.1183G>A/ c.1979C>T leads to SEMD, the phenotype of 1979C>T in previous study was SSOAOD. *The phenotype of heterozygous mutant was SSOAOD, and phenotype of the homozygous mutant was SEMD.


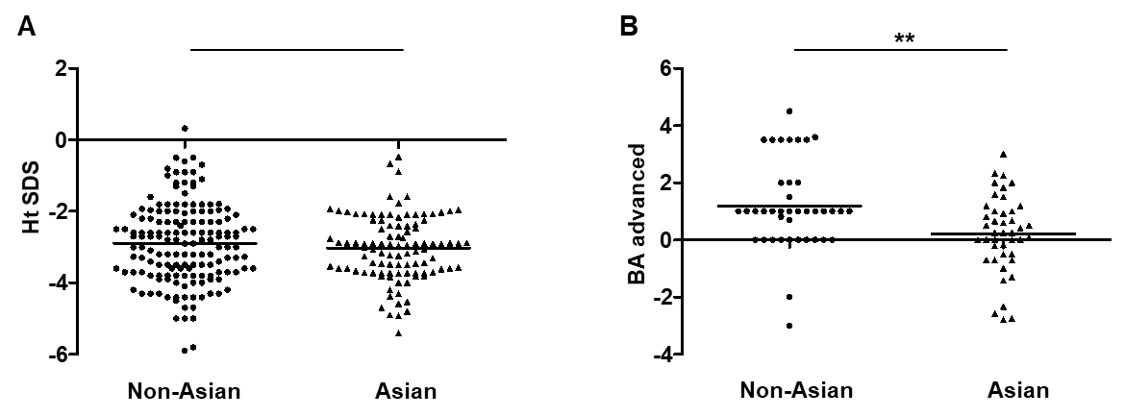


**Fig S5. Analysis of HtSDS and BA-CA between non-Asian and Asian populations in SSOAOD patients.** (A) Comparison of HtSDS between non-Asian and Asian populations. (B) Comparison of BA-CA between non-Asian and Asian populations. Abbreviation: * P<0.05, **P<0.01. Ht SDS: height standard deviation, BA: bone age, CA: Biological age.

**
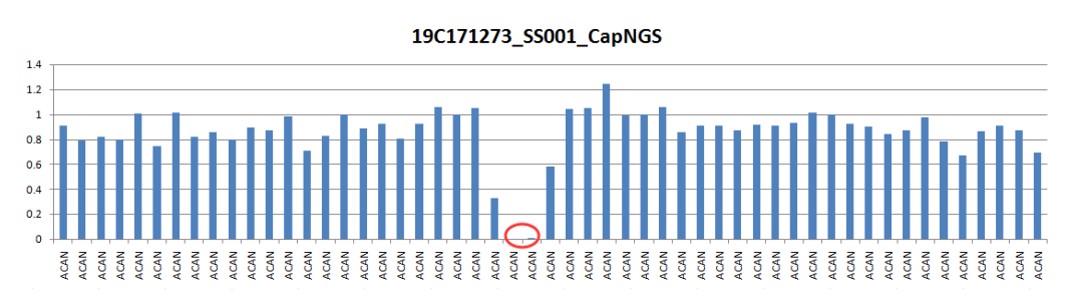
Fig S6. Coverage analysis from exome sequencing data encompassing the homozygous exon12 deletion in ACAN of the proband 17**
