# Supplementary material for: The Spectrum of ACAN Gene Mutations in a Selected Chinese Cohort of Short Stature: Genotype-Phenotype Correlation
Source: Front Genet. 2022 May 10;13:891040. doi: 10.3389/fgene.2022.891040 (PMC9127616; doi:10.3389/fgene.2022.891040)
Supplement: Supplementary file 2 [file DataSheet3.DOCX]

**Supplementary Methods**

**Growth hormone stimulation test**

Growth hormone stimulation tests was performed by two kinds of drugs (arginine 0.5 g/kg, intravenously, and clonidine 4 μg/kg, orally) in two days. GH levels were measured at standard intervals during the tests (0, 30, 60, 90, 120 min). According to current guidelines^1-2^, the peak growth hormone levels more than 10 ng/ml are defined as normal, peak growth hormone levels between 5 and 10 ng/ml are defined as partial growth hormone deficiency, and the peak growth hormone levels less than 5 ng/ml are defined as complete growth hormone deficiency.

**Therapeutic indication and dose of rhGH**

Indications for rhGH include GHD, Turner syndrome, Prader-Willi syndrome, chronic renal failure, small for gestational age, and idiopathic short stature. The recommended initial dose of rhGH is 0.1-0.15 IU/kg, for pubertal patients and patients with Turner syndrome, SGA, ISS, PGHD, the dose can be increased to 0.15-0.2 IU/kg, It should be emphasized that the dose range of growth hormone is relatively wide and should be adjusted individually according to clinical needs and efficacy^2^.

**Defining growth response of GH treatment**

In this study, we calculated height SDS based on Chinese growth curves ^3^. To evaluate the growth response of GH treatment, we mainly used height SDS (including the first-year and annualized height SDS) and referred to height velocity. We regarded patients with a first-year delta height SDS larger than 0.3–0.5 or a first-year height velocity increase larger than 3 cm/year as of good response^4^. If the annualized height SDS of these patient was smaller than 0.3, it was defined as of modest response. If the response was worse than above measurements, it was defined as of poor response. Patients treated for less than one year were not evaluated.

**Bone age**

Bone age was assessed using the G&P methods by independent manual rating of two observers, then take an average.

**Sanger sequencing**

Genomic DNA was routinely extracted using a DNA isolation kit (Tiangen, China) according to the manufacturer’s instructions. All the exonic sequences and intron–exon boundaries of *ACAN* (Ensembl Accession ENST00000379374.5) were amplified using polymerase chain reaction (PCR) and primers designed in Primer Premier 5.0 (Table 1). The PCR products were gel- and column-purified and directly sequenced. The purified PCR fragments were sequenced using Big Dye Terminator (Applied Biosystems, Foster City, CA, USA) on an ABI 3130 genetic analyzer (Applied Biosystems).All variants were denoted based on the NCBI reference sequence for *ACAN* (NM_000444.6).

**Minigene Assay/pSPL3 splicing assay**

To confirm the probable splice mutation that affected an intron–exon junction site, in *vitro* analysis was performed using a minigene splicing assay based on the pSPL3 exon trapping vector. Fragments with wild-type or mutant alleles containing the exon of interest flanked by and upstream intronic sequence and downstream intronic sequence were cloned into the splicing vector pSPL3 using specific primers linking the XhoI and BamHI restriction enzyme sites. All of the plasmids were sequenced to confirm the distinct mutations and absence of off-target mutations in the constructs. Plasmids were transfected into HEK293 cells, and mRNA was reverse transcribed.

**Table 1 Primers used for amplification and sequencing of the coding regions of *ACAN* gene**

| ***ACAN* region** | **Forward primer (5′→3′)** | **Reverse primer (5 ′→3 ′ )** |
| --- | --- | --- |
| Exon 1 | GGTTTCCCTTTGCGCTCG | CGGCACACATAGCTCTAGGG |
| Exon 2 | ATGATTCCAGGTCCTTGGGT | GACCAGTAGCACCAGGTTCA |
| Exon 3 | CGGATTTCGCTCTCTCAGGA | TCCATGTCACAGAGGAAGCA |
| Exon 4 | CAGACCAGCCAGTTCCCTAA | TCATTGAGCTGCTGTTTGGC |
| Exon 5 | ACGGGAGGAGGATTCAAAGG | CAGGTGTCAGTGGGGAGATG |
| Exon 6 | GCAGACATATGGGACCAGGA | ACCCTCCCTTTCCAAGATCC |
| Exon 7 | CTCATCTCCAGCCCACTCC | GCCTTCAACTGCGGGAATC |
| Exon 8 | CAAGTGGGATTTCAGCCTGC | TCCTTAAGCCTGACCTCTTCA |
| Exon 9 | CTTGCTGCATAAGGGGCTTT | CTTTGTATCGCCCTGTCAGC |
| Exon 10 | TGGTGAGGAGGGTTAGAGGA | TCTTGCTCTGATGGATGGGA |
| Exon 11 | CCCTAGCTCCCCTCAAGAAG | AAACAGCACAAATCCCAGGG |
| Exon 12 |  |  |
| ① | CACGTTGCTGAGCTCTTGAG | GAAGTCCACTGAGATCCCCA |
| ② | TGCCTGTGGAAAGTGGACTA | TAGTCCACTTTCCACAGGCA |
| ③ | TTCTGGAGAGGTTGTAGAGACT | CGCTAAGCTCAGTCACTCCA |
| ④ | GAGCTAGTGGACTCCCTTCA | ACCCTCCACGAACTCAGAAG |
| ⑤ | TCAGTGGTGCTCATTCTGGA | ACGTTACAGATGAGGCTCAGA |
| Exon 13 | CCTCATGCCCCAACTTTGAG | GTCATCATGCAGCTCCTTCC |
| Exon 14 | GTGAACGCAGGAACCTATGC | CCCAAATCTGCTGCCTTCTG |
| Exon 15 | CACCTCCTTTCCTCCTCCAT | CTGTCTGCATGGGGTTTGAA |
| Exon 16 | ATGATGAAGAGGCTCCACGG | TGTGACCTTCATGTGCTTGC |
| Exon 17 | CATGAAAACGTCCAGGGCTC | CTTCCCTTGCTCAACCCTGT |
| Exon 18 | CCTGAGTCCTGGTTTCCACA | CAAAACCTGTCTCCACTGCC |

**References：**

1. Grimberg, A.; DiVall, S. A.; Polychronakos, C.; Allen, D. B.; Cohen, L. E.; Quintos, J. B.; Rossi, W. C.; Feudtner, C.; Murad, M. H., Guidelines for Growth Hormone and Insulin-Like Growth Factor-I Treatment in Children and Adolescents: Growth Hormone Deficiency, Idiopathic Short Stature, and Primary Insulin-Like Growth Factor-I Deficiency. *Hormone research in paediatrics* **2016,** *86* (6), 361-397.

2. The Subspecialty Group of Endocrinologic; Hereditary and Metabolic Diseases; The Society of Pediatrics; Chinese Medical Association, [Guidelines for diagnosis and treatment of children with short stature]. *Zhonghua er ke za zhi* **2008,** *46* (24), 20-21.

3. Li, H.; Ji, C. Y.; Zong, X. N.; Zhang, Y. Q., [Height and weight standardized growth charts for Chinese children and adolescents aged 0 to 18 years]. *Zhonghua er ke za zhi = Chinese journal of pediatrics* **2009,** *47* (7), 487-92.

4. Cohen, P.; Rogol, A. D.; Deal, C. L.; Saenger, P.; Reiter, E. O.; Ross, J. L.; Chernausek, S. D.; Savage, M. O.; Wit, J. M., Consensus statement on the diagnosis and treatment of children with idiopathic short stature: a summary of the Growth Hormone Research Society, the Lawson Wilkins Pediatric Endocrine Society, and the European Society for Paediatric Endocrinology Workshop. *The Journal of clinical endocrinology and metabolism* **2008,** *93* (11), 4210-7.
