# Supplementary material for: The Spectrum of ACAN Gene Mutations in a Selected Chinese Cohort of Short Stature: Genotype-Phenotype Correlation
Source: Front Genet. 2022 May 10;13:891040. doi: 10.3389/fgene.2022.891040 (PMC9127616; doi:10.3389/fgene.2022.891040)
Supplement: Supplementary file 3 [file DataSheet2.DOCX]

**Supplementary Table 3. Comparison of clinical data between two groups with truncating and non-truncating variants**

|  | **Non-truncating variants** | **Truncating variants** | **P-value** |
| --- | --- | --- | --- |
| **N** | 32 | 62 |  |
| **Age(years)** | 10.8 ± 8.7 | 9.0 ± 4.3 | 0.661 |
| **Height SDs** | -2.7 ± 0.9 | -3.1 ± 0.9 | 0.089 |
| **BA-CA (years)** | 0.2 ± 1.5 | 0.9 ± 1.5 | **0.046** |
| **Sex** |  |  | 0.484 |
| Male | 17 (53.1%) | 37 (60.7%) |  |
| Female | 15 (46.9%) | 24 (39.3%) |  |
| **SGA** |  |  | 0.563 |
| no | 9 (64.3%) | 19 (73.1%) |  |
| yes | 5 (35.7%) | 7 (26.9%) |  |
| **BA advanced** |  |  | 0.092 |
| no | 15 (50.0%) | 18 (31.6%) |  |
| yes | 15 (50.0%) | 39 (68.4%) |  |
| **Nationality** |  |  | 0.148 |
| Non-Asian | 20 (62.5%) | 29 (46.8%) |  |
| Asian | 12 (37.5%) | 33 (53.2%) |  |
| **De novo** |  |  | 0.295 |
| no | 28 (100.0%) | 51 (92.7%) |  |
| yes | 0 (0.0%) | 4 (7.3%) |  |
| **Location** |  |  | **0.04** |
| E2 | 0 (0.0%) | 3 (4.8%) |  |
| E3 | 3 (9.4%) | 2 (3.2%) |  |
| E4 | 3 (9.4%) | 5 (8.1%) |  |
| E5 | 1 (3.1%) | 2 (3.2%) |  |
| E6 | 4 (12.5%) | 3 (4.8%) |  |
| E7 | 0 (0.0%) | 6 (9.7%) |  |
| E8 | 0 (0.0%) | 2 (3.2%) |  |
| E9 | 1 (3.1%) | 2 (3.2%) |  |
| E10 | 5 (15.6%) | 5 (8.1%) |  |
| E11 | 3 (9.4%) | 2 (3.2%) |  |
| E12 | 1 (3.1%) | 17 (27.4%) |  |
| E13 | 0 (0.0%) | 1 (1.6%) |  |
| E14 | 2 (6.2%) | 0 (0.0%) |  |
| E15 | 3 (9.4%) | 2 (3.2%) |  |
| E16 | 3 (9.4%) | 5 (8.1%) |  |
| E17 | 2 (6.2%) | 0 (0.0%) |  |
| E18 | 1 (3.1%) | 0 (0.0%) |  |
| I10 | 0 (0.0%) | 2 (3.2%) |  |
| I2 | 0 (0.0%) | 1 (1.6%) |  |
| I7 | 0 (0.0%) | 1 (1.6%) |  |
| I9 | 0 (0.0%) | 1 (1.6%) |  |
| **Protein domain** |  |  | **0.01** |
| signal peptide | 0 (0.0%) | 3 (4.8%) |  |
| G1 | 11 (34.4%) | 12 (19.4%) |  |
| IGD | 0 (0.0%) | 5 (8.1%) |  |
| G2 | 6 (18.8%) | 10 (16.1%) |  |
| KS | 3 (9.4%) | 5 (8.1%) |  |
| CS | 1 (3.1%) | 14 (22.6%) |  |
| G3 | 11 (34.4%) | 8 (12.9%) |  |
| splicing | 0 (0.0%) | 5 (8.1%) |  |

**Abbreviations: CS, chondroitin sulfate attachment domain; G1, globular domain 1; G2, globular domain 2; G3, globular domain 3; IGD, interglobular domain; KS, keratin sulfate attachment domain.**

**Supplementary Table 4. Comparison of clinical data between Asian and non-Asian groups**

|  | **Non-Asian** | **Asian** | **P-value** |
| --- | --- | --- | --- |
| **N** | 49 | 45 |  |
| **Age(years)** | 11.1 ± 8.8 | 8.8 ± 4.0 | 0.3 |
| **Height SDs** | -2.9 ± 0.9 | -3.0 ± 0.8 | 0.812 |
| **BA-CA (years)** | 1.2 ± 1.6 | 0.2 ± 1.3 | **0.003** |
| **Sex** |  |  | 0.431 |
| Male | 26 (54.2%) | 28 (62.2%) |  |
| Female | 22 (45.8%) | 17 (37.8%) |  |
| **SGA** |  |  | 0.112 |
| no | 11 (57.9%) | 17 (81.0%) |  |
| yes | 8 (42.1%) | 4 (19.0%) |  |
| **BA advanced** |  |  | 0.235 |
| no | 14 (31.8%) | 19 (44.2%) |  |
| yes | 30 (68.2%) | 24 (55.8%) |  |
| **De novo** |  |  | 0.635 |
| no | 45 (93.8%) | 34 (97.1%) |  |
| yes | 3 (6.2%) | 1 (2.9%) |  |
| **Mutation type** |  |  | 0.14 |
| small deletion | 0 (0.0%) | 1 (2.2%) |  |
| frameshift | 9 (18.4%) | 17 (37.8%) |  |
| missense | 20 (40.8%) | 11 (24.4%) |  |
| nonsense | 18 (36.7%) | 13 (28.9%) |  |
| splicing | 2 (4.1%) | 3 (6.7%) |  |
| **Truncating variants** |  |  | 0.148 |
| no | 20 (40.8%) | 12 (26.7%) |  |
| yes | 29 (59.2%) | 33 (73.3%) |  |
| **Location** |  |  | 0.114 |
| E2 | 2 (4.1%) | 1 (2.2%) |  |
| E3 | 5 (10.2%) | 0 (0.0%) |  |
| E4 | 4 (8.2%) | 4 (8.9%) |  |
| E5 | 1 (2.0%) | 2 (4.4%) |  |
| E6 | 4 (8.2%) | 3 (6.7%) |  |
| E7 | 3 (6.1%) | 3 (6.7%) |  |
| E8 | 1 (2.0%) | 1 (2.2%) |  |
| E9 | 3 (6.1%) | 0 (0.0%) |  |
| E10 | 2 (4.1%) | 8 (17.8%) |  |
| E11 | 1 (2.0%) | 4 (8.9%) |  |
| E12 | 9 (18.4%) | 9 (20.0%) |  |
| E13 | 0 (0.0%) | 1 (2.2%) |  |
| E14 | 2 (4.1%) | 0 (0.0%) |  |
| E15 | 2 (4.1%) | 3 (6.7%) |  |
| E16 | 7 (14.3%) | 1 (2.2%) |  |
| E17 | 1 (2.0%) | 1 (2.2%) |  |
| E18 | 0 (0.0%) | 1 (2.2%) |  |
| I10 | 2 (4.1%) | 0 (0.0%) |  |
| I2 | 0 (0.0%) | 1 (2.2%) |  |
| I7 | 0 (0.0%) | 1 (2.2%) |  |
| I9 | 0 (0.0%) | 1 (2.2%) |  |
| **Protein domain** |  |  | 0.624 |
| signal peptide | 2 (4.1%) | 1 (2.2%) |  |
| G1 | 14 (28.6%) | 9 (20.0%) |  |
| IGD | 2 (4.1%) | 3 (6.7%) |  |
| G2 | 7 (14.3%) | 9 (20.0%) |  |
| KS | 2 (4.1%) | 6 (13.3%) |  |
| CS | 8 (16.3%) | 7 (15.6%) |  |
| G3 | 12 (24.5%) | 7 (15.6%) |  |
| splicing | 2 (4.1%) | 3 (6.7%) |  |

**Abbreviations: CS, chondroitin sulfate attachment domain; G1, globular domain 1; G2, globular domain 2; G3, globular domain 3; IGD, interglobular domain; KS, keratin sulfate attachment domain.**
